# Calcium-Regulated Self-Assembly of Zwitterionic/Cationic Surfactant–Counterion Complexes: Molecular Dynamics Insights into the Suppression of Wormlike Micelle Growth

**DOI:** 10.3390/molecules31142490

**Published:** 2026-07-16

**Authors:** Yazhou Wang, Fajun Guo, Xiaonan Feng, Hongmei Wang, Zhao Xue, Shuai Zhang, Mingwei Gao

**Affiliations:** 1 PetroChina Huabei Oilfield Company, Renqiu 062552, China; 2 State Key Laboratory of Deep Oil and Gas, China University of Petroleum (East China), Qingdao 266580, China; 3 Key Laboratory of Unconventional Oil & Gas Development, School of Petroleum Engineering, China University of Petroleum (East China), Ministry of Education, Qingdao 266580, China

**Keywords:** wormlike micelles, calcium-regulated self-assembly, zwitterionic surfactants, cationic surfactants, counterion complexes, molecular dynamics simulation, hydration effects, hydrophobic-chain packing

## Abstract

Wormlike micelles provide the structural basis for the viscoelasticity of clean fracturing fluids, yet the molecular mechanism by which calcium-containing brine regulates their growth remains insufficiently understood. In this work, all-atom molecular dynamics simulations were performed to investigate the calcium-regulated self-assembly of an EAHSB/EHAC/NaPts surfactant–counterion system. In the absence of Ca^2+^, dispersed surfactant and counterion molecules first formed small spherical aggregates, which subsequently fused into larger micelles, rod-like intermediates, and finally wormlike micelles. Increasing the local surfactant/counterion loading further promoted one-dimensional micellar growth and network-like association. When Ca^2+^ was introduced, the assembly process became slower and the final aggregate morphology shifted from elongated wormlike micelles to smaller spherical aggregates. Radial distribution functions and coordination-number analysis show that Ca^2+^ redistributes charged-group correlations within the mixed surfactant/counterion assemblies. Hydration analysis indicates enlarged polar hydration shells, whereas hydrophobic-tail hydration and gauche-defect probabilities reveal enhanced tail exposure and looser chain packing. These results demonstrate that Ca^2+^ suppresses wormlike micelle growth through coupled electrostatic redistribution, interfacial hydration, and hydrophobic-chain disorder, thereby favoring high-curvature spherical aggregates over extended wormlike networks.

## 1. Introduction

Wormlike micelles (WLMs) are one-dimensional supramolecular aggregates formed by the reversible self-assembly of amphiphilic molecules in aqueous solution. Because they can continuously break and recombine, WLMs are often regarded as “living polymers” and can form transient entangled networks when their contour length and concentration are sufficiently high [1,2,3]. These dynamic networks impart high zero-shear viscosity, elasticity, and shear-thinning behavior to viscoelastic surfactant fluids, while avoiding the covalent macromolecular backbones and solid residues associated with conventional polymer-thickened systems [3,4]. Such features make WLM-based fluids attractive for clean hydraulic fracturing, where the working fluid must provide sand-carrying capacity during pumping and allow efficient cleanup after stimulation [4]. However, the macroscopic rheology of these fluids is ultimately governed by microscopic self-assembly processes, including the formation of small micelles, their coalescence into larger aggregates, and their subsequent elongation into cylindrical or wormlike structures.

Mixed ionic surfactant systems provide an effective route for promoting WLM growth because electrostatic interactions can be tuned by combining oppositely charged, zwitterionic, and counterion-containing species [5,6,7,8]. In such systems, ion-pair-like associations between charged headgroups and counterions can reduce headgroup repulsion, modify the effective interfacial curvature, and favor the transition from spherical micelles to elongated aggregates [5,6,7]. According to classical packing-parameter theory, spherical micelles are favored when the effective headgroup area is large relative to the hydrophobic tail volume, whereas cylindrical or wormlike micelles become more favorable when the interfacial curvature is reduced [9]. Therefore, WLM formation is controlled by a coupled balance among charged-group association, charge screening, counterion organization, hydrophobic-chain packing, and interfacial hydration. Hydration is particularly important because water molecules around ionic or zwitterionic headgroups can change the effective headgroup area and thereby shift the preferred aggregate curvature [10].

The same sensitivity that enables the formation of WLMs also makes their growth strongly dependent on electrolyte conditions. Low or moderate salt levels may screen electrostatic repulsion and assist micellar elongation, whereas excessive ionic strength or specific multivalent ions can reorganize charged groups, compete with organic counterions, and alter hydration at the micellar interface [11,12,13,14]. Divalent cations such as Ca^2+^ are especially relevant for oilfield brines because they combine strong electrostatic influence with pronounced hydration and ion-specific effects [11,13]. Previous experimental and simulation studies have shown that the valency, radius, and hydration characteristics of cations can significantly affect the morphology and rheology of ionic or zwitterionic surfactant assemblies [11,12,13,14]. Thus, Ca^2+^ may not act simply as a nonspecific charge-screening agent; instead, it may regulate self-assembly by simultaneously changing local electrostatic correlations, headgroup hydration, and hydrophobic-core organization.

Molecular dynamics (MD) simulation provides a useful tool for resolving these molecular-level processes because it can track aggregate growth, ion distributions, water organization, and chain conformations directly. Previous simulations have examined mixed cationic/anionic WLMs, the transformation between linear and branched micelles, and the influence of Ca^2+^ and Mg^2+^ on surfactant solutions [11,15,16]. More recent molecular simulation studies have also emphasized the value of atomistic or coarse-grained approaches for connecting surfactant self-assembly with interfacial adsorption, micellization, and formulation performance [17,18]. From an application perspective, the stability of wormlike micelles in Ca^2+^-rich brines is particularly important for clean fracturing fluids. In many oilfield formations, divalent cations can interact with ionic or zwitterionic surfactant headgroups, alter counterion organization, and consequently change the micellar morphology and viscoelastic response. Therefore, clarifying how Ca^2+^ affects the growth of wormlike micelles is essential for the molecular design of salt-tolerant viscoelastic surfactant fluids. The EAHSB/EHAC/NaPts system was selected as a representative zwitterionic/cationic surfactant–counterion system. EAHSB provides both sulfonate and quaternary ammonium groups, EHAC contributes cationic headgroups and long hydrophobic chains, and Pts^−^ acts as an organic counterion that can regulate headgroup association and interfacial packing. This combination allows the roles of charged-group association, counterion organization, hydration, and hydrophobic-chain packing to be examined in a unified molecular framework. Nevertheless, the molecular mechanism by which Ca^2+^ regulates the self-assembly of zwitterionic/cationic surfactant–counterion complexes remains insufficiently understood. In particular, it is still unclear how calcium ions suppress the growth of long wormlike micelles and shift the assembly endpoint toward smaller, higher-curvature aggregates.

In this work, all-atom MD simulations were used to investigate the calcium-regulated self-assembly of an EAHSB/EHAC/NaPts surfactant–counterion system. First, concentration-dependent simulations were performed to clarify how dispersed surfactant and counterion molecules evolve into spherical micelles, rod-like intermediates, and wormlike micelles. Then, Ca^2+^ was introduced at different ratios to examine how calcium-containing brine changes the assembly rate, final aggregate morphology, charged-group correlations, hydration behavior, and hydrophobic-tail conformation. Radial distribution functions, coordination numbers, hydration numbers, and gauche-defect probabilities were combined to establish a molecular-level picture of Ca^2+^-regulated micellar growth. The results show that Ca^2+^ suppresses wormlike micelle formation through coupled electrostatic redistribution, enhanced interfacial hydration, and looser hydrophobic-chain packing, thereby favoring smaller spherical aggregates rather than extended wormlike networks.

## 2. Results and Discussion

### 2.1. Concentration-Dependent Self-Assembly of EAHSB/EHAC/NaPts

The self-assembly simulations were first performed at a fixed EAHSB:EHA^+^:Pts^−^ molar ratio of 1:1:1, while the total number of surfactant/counterion species was varied. As summarized in Table 1, each simulation box contained 30,000 water molecules. Because a finite periodic simulation box cannot exchange surfactant molecules or counterions with the surrounding bulk phase, these systems are treated as local-enrichment models rather than direct reproductions of macroscopic bulk concentrations. The series was therefore designed to compare how different local surfactant/counterion loadings affect aggregate growth and the formation of elongated micelles.

**Figure 1:**
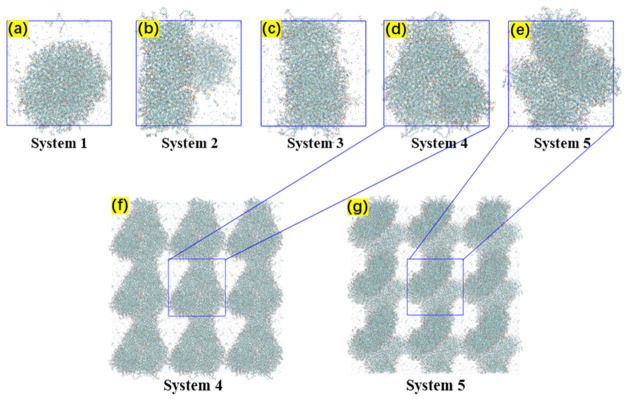
Equilibrium aggregate morphologies of the calcium-free EAHSB/EHA^+^/Pts^−^ systems at a fixed molar ratio of 1:1:1. (**a**–**e**) Systems 1–5 with increasing local surfactant/counterion loading. (**f**,**g**) Periodic-image views of systems 4 and 5 showing extended network-like micellar association. Water molecules and simple ions are omitted for clarity.

As shown in Figure 1a–e, the equilibrium aggregates changed from compact spherical or ellipsoidal micelles to long cylindrical micelles as the local loading increased. In the high-loading systems, the periodic-image snapshots in Figure 1f–g showed that neighboring wormlike micelles were not isolated objects; they formed an extended, interwoven network. This morphology is consistent with the known concentration sensitivity of wormlike micelles, where one-dimensional growth and entanglement give rise to viscoelastic behavior in solution [1,2,5]. This concentration-dependent transition is also consistent with previous studies of mixed ionic wormlike micelles, in which increased surfactant loading and strengthened intermolecular association promote one-dimensional micellar growth and network formation [5,6,7]. Similar behavior has been observed in simulations of mixed cationic/anionic wormlike micelles, where electrostatic association between oppositely charged species reduces effective headgroup repulsion and favors cylindrical or wormlike aggregates [15,16]. Therefore, the morphology evolution observed here follows the general self-assembly behavior of mixed ionic surfactant systems.

### 2.2. Time-Resolved Pathway from Spherical Aggregates to Wormlike Micelles

**Figure 2:**
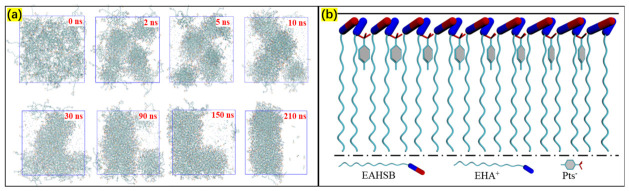
Time-resolved self-assembly pathway and proposed molecular arrangement of System 3. (**a**) Simulation snapshots at 0, 2, 5, 10, 30, 90, 150, and 210 ns. (**b**) Schematic representation of the proposed EAHSB/EHA^+^/Pts^−^ molecular arrangement within the aggregate. Water molecules and simple ions are omitted for clarity.

System 3 was selected to follow the assembly pathway in more detail because it represents an intermediate local surfactant/counterion loading in the calcium-free concentration series and has the same EAHSB/EHAC/Pts^−^ composition as the calcium-free reference system used for the Ca^2+^-containing simulations. Therefore, System 3 provides a suitable baseline for resolving the intrinsic self-assembly pathway and for comparison with the subsequent Ca^2+^-regulated assembly behavior. As shown in Figure 2a, the dispersed surfactant/counterion molecules rapidly formed small spherical aggregates within 2 ns. These small micelles then fused into larger spherical aggregates by 10 ns. At 30 ns, rod-like intermediates appeared, and by 90 ns the first wormlike micelles were visible while some spherical micelles remained free in solution. At the late stage of the trajectory, the residual spherical micelles merged into the elongated aggregate, giving a single long and relatively straight wormlike micelle at 150 ns. The final micelle had a diameter of approximately 5 nm. Block-average analysis of the maximum aggregate size showed values of 175 and 180 molecules for the 90–150 ns and 150–210 ns windows, respectively, corresponding to a variation of less than 3%. This result indicates that micellar growth had reached a stable plateau within the 210 ns trajectory. In the present work, the morphology assignment was mainly based on time-resolved aggregate snapshots, together with the subsequent RDF, coordination-number, hydration-number, and hydrophobic-tail conformation analyses. These analyses were used to identify the dominant morphology transition and its molecular origin. Additional quantitative descriptors, such as radius of gyration, sphericity, shape factor, and principal moments of inertia, would further strengthen future large-scale morphology analyses, especially for aggregates extending across periodic boundaries. Therefore, the present morphology assignment was interpreted together with RDF, coordination-number, hydration-number, and hydrophobic-tail conformation analyses rather than from snapshots alone.

This sequence suggests a fusion-controlled growth pathway: small micelles form first, larger aggregates follow, and wormlike morphology appears only after repeated coalescence. The final morphology is therefore governed by both the local abundance of the surfactant/counterion species and the ability of the growing aggregates to maintain low-curvature packing.

The self-assembly behavior is closely related to the chemical structures of EAHSB, EHAC, and Pts^−^. EAHSB contains both a sulfonate group and a quaternary ammonium group, providing zwitterionic charged sites for local electrostatic association. EHAC/EHA^+^ contains a cationic quaternary ammonium headgroup and a long hydrophobic chain, contributing to both charged-group association and hydrophobic-core formation. Pts^−^ acts as an organic counterion and is distributed near quaternary ammonium groups, thereby reducing effective headgroup repulsion and assisting low-curvature packing. Meanwhile, the long hydrophobic chains of EAHSB and EHAC pack together to form the micellar core. Therefore, the balance among charged-group association, counterion organization, interfacial hydration, and hydrophobic-chain packing governs the transition from spherical aggregates to wormlike micelles. Based on the time-resolved snapshots and the charged-group correlation analysis discussed below, a schematic representation of the proposed molecular arrangement is shown in Figure 2b. This schematic is intended to illustrate the possible organization of EAHSB, EHAC/EHA^+^, and Pts^−^ within the aggregate, rather than to serve as an independent trajectory analysis. The sulfonate group of EAHSB appears to associate with the positively charged quaternary ammonium group of EHAC/EHA^+^, while Pts^−^ counterions are distributed near the quaternary ammonium group of EAHSB and the amide-containing interfacial region of EHAC. EHAC/EHA^+^ is located between EAHSB-rich regions and contributes to both charged-group association and local charge screening. Meanwhile, the hydrophobic chains of EAHSB and EHAC pack together to form the micellar core. The behavior resembles mixed ionic surfactant systems in which oppositely charged species can form ion-pair amphiphiles and alter aggregate curvature [8].

It should be noted that the 210 ns trajectory was used to identify the relative self-assembly pathway of System 3 under the present finite-box conditions, rather than to fully capture long-time wormlike micelle entanglement or rheological relaxation. Within this simulation window, the calcium-free system showed a clear transition from dispersed molecules to spherical aggregates, rod-like intermediates, and an elongated wormlike aggregate. Therefore, the 210 ns trajectory was considered adequate for comparing the relative morphology evolution discussed in this work, although longer-time and larger-scale simulations are still required to fully resolve slow wormlike micelle growth, entanglement, and network relaxation.

### 2.3. Ca^2+^ Regulates Both the Rate and Endpoint of Self-Assembly

The calcium-containing simulations fixed the numbers of EAHSB, EHA^+^, and Pts^−^ at 180 each, while Ca^2+^ was introduced at Ca^2+^:EAHSB:EHA^+^:Pts^−^ ratios of 0:1:1:1, 10:1:1:1, and 20:1:1:1. The full compositions are listed in Table 2. To help readers understand the concentration level of the finite-box systems, the nominal Ca^2+^ molarity was also included in Table 2. These values were used only to describe the local composition of the simulation boxes. Because the boxes represent locally enriched micellar domains and cannot exchange surfactants, counterions, or salts with the surrounding bulk phase, the nominal concentrations should not be regarded as direct macroscopic formulation concentrations. The 10:1:1:1 system was selected to represent a calcium-rich brine condition relevant to fracturing-fluid cleanup tests, whereas the 20:1:1:1 system was used to amplify calcium effects and identify the limiting trend. These Ca^2+^-containing systems should be interpreted as local-enrichment models rather than direct reproductions of bulk brine concentrations. Na^+^ and Cl^−^ ions were added to maintain charge neutrality and the designed ionic composition.

**Figure 3:**
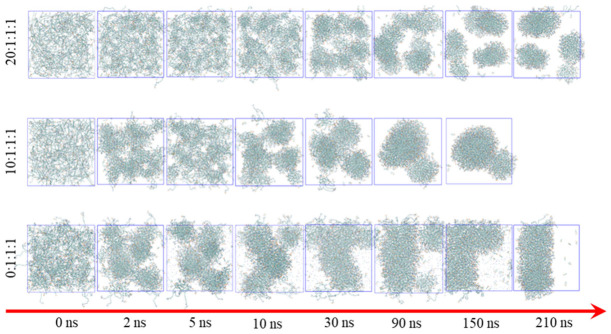
Time-resolved morphology evolution of EAHSB/EHA^+^/Pts^−^ systems at Ca^2+^:EAHSB:EHA^+^:Pts^−^ ratios of 0:1:1:1, 10:1:1:1, and 20:1:1:1. The snapshots were collected at 0, 2, 5, 10, 30, 90, 150, and 210 ns. Water molecules and simple ions are omitted for clarity.

As shown in Figure 3, the calcium-free system again assembled into wormlike micelles. In the Ca^2+^-containing systems, however, the final aggregates were predominantly spherical, and their apparent size decreased as the Ca^2+^ loading increased. Calcium also slowed the assembly process. In the absence of Ca^2+^, a relatively large spherical aggregate was already present at 2 ns. In the Ca^2+^-containing systems, aggregates of comparable size appeared only after about 10 ns and 30 ns, respectively. These results indicate that Ca^2+^ regulates both the kinetic pathway and the final morphological endpoint of surfactant/counterion self-assembly. The molecular origin of this calcium-regulated behavior is further analyzed in the following sections through charged-group correlations, coordination numbers, hydration numbers, and hydrophobic-tail conformational descriptors. These analyses show that the Ca^2+^-containing environment reorganizes the interfacial charged-group correlations, enhances polar hydration, and weakens compact hydrophobic-chain packing, which together explain the slower aggregate growth and the shift from wormlike micelles to smaller spherical aggregates.

The same simulation length was used for all calcium-free and Ca^2+^-containing systems, allowing the relative effect of Ca^2+^ on aggregate growth and final morphology to be compared under identical simulation conditions. Therefore, the observed differences should be interpreted as relative morphology trends within the 210 ns simulation window.

### 2.4. Charged-Group Correlations and Coordination Numbers

**Figure 4:**
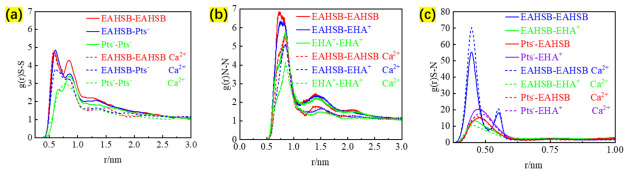
Radial distribution functions of representative charged-group pairs in the calcium-free and Ca^2+^-containing systems. (**a**) RDFs involving sulfonate-related group pairs, including EAHSB_S–EAHSB_S, EAHSB_S–Pts_S, and Pts_S–Pts_S. (**b**) RDFs involving quaternary-ammonium-related group pairs, including EAHSB_N–EAHSB_N, EAHSB_N–EHAC_N, and EHAC_N–EHAC_N. (**c**) RDFs between Pts^−^ counterions and surfactant charged groups, including Pts_S–EAHSB_S, Pts_S–EAHSB_N, and Pts_S–EHAC_N. Solid and dashed lines represent the 0:1:1:1 and 20:1:1:1 systems, respectively.

As shown in Figure 4, RDFs were used to characterize how Ca^2+^ altered local charged-group spatial correlations within the aggregates. At the Ca^2+^:EAHSB:EHA^+^:Pts^−^ ratio of 20:1:1:1, several RDF peaks associated with sulfonate–sulfonate, quaternary-ammonium–quaternary-ammonium, and quaternary-ammonium–sulfonate group pairs decreased in intensity. These changes indicate a redistribution of local charged-group organization rather than the complete removal of electrostatic association. In contrast, the EAHSB sulfonate-EAHSB quaternary ammonium peak increased, while the EAHSB sulfonate-EHAC quaternary ammonium peak shifted to shorter distance but became weaker.

The 20:1:1:1 system was selected for the detailed correlation analysis because it showed the most pronounced calcium-regulated morphological shift. As summarized in Table 3, the coordination numbers provide a quantitative measure of the average number of neighboring charged sites within the first coordination shell. Several charged-group correlations decreased in the Ca^2+^-containing system: EAHSB_S-EAHSB_S decreased from 0.97 to 0.72, EAHSB_N-EAHSB_N from 2.20 to 1.76, EAHSB_N-EHAC_N from 2.20 to 1.81, EHAC_N-EHAC_N from 1.90 to 1.55, EHAC_N-Pts_S from 2.46 to 2.16 and Pts_S-Pts_S from 0.41 to 0.05. Contacts between EAHSB_S and EHAC_N also decreased from 1.69 to 1.40, and EAHSB_S-Pts_S decreased from 1.08 to 0.92. The main exception was the EAHSB_S-EAHSB_N contact, which increased from 2.95 to 3.62, while EAHSB_N-Pts_S remained nearly unchanged. Thus, Ca^2+^ does not eliminate electrostatic organization within the aggregates; instead, it redistributes local charged-group correlations, strengthening some EAHSB-centered correlations while weakening several intermolecular correlations that connect different surfactant/counterion species. In Figure 4a, the weakened RDF peaks of sulfonate-related pairs indicate that the Ca^2+^-containing environment changes the local organization of sulfonate groups and Pts^−^ counterions. In Figure 4b, the reduced peak intensities of quaternary-ammonium-related pairs suggest weaker intermolecular association among cationic headgroups. In Figure 4c, the changes in Pts^−^-related RDFs show that the spatial correlation between organic counterions and surfactant charged groups is also redistributed. These RDF changes indicate that the Ca^2+^-containing environment does not simply produce nonspecific charge screening, but redistributes the local charged-group organization within the mixed surfactant/counterion aggregates.

These coordination-number changes provide evidence that the Ca^2+^-containing environment reorganizes the interfacial charged layer of the mixed aggregates. In the Ca^2+^-containing system, several intermolecular correlations connecting different surfactant/counterion species were weakened, including EAHSB_S-EHAC_N, EAHSB_N-EHAC_N, EHAC_N-EHAC_N, EHAC_N-Pts_S, and Pts_S-Pts_S. These decreases indicate that the continuous mixed charged-group association between EAHSB, EHAC, and Pts^−^ becomes less favorable in the Ca^2+^-containing environment. In contrast, the increase in EAHSB_S-EAHSB_N correlation suggests a more localized EAHSB-centered association. Therefore, Ca^2+^ does not simply promote nonspecific charge screening; rather, the Ca^2+^-containing environment redistributes local electrostatic organization at the micellar interface. This redistribution weakens the intermolecular associations required for sustained one-dimensional micellar growth and provides a molecular basis for the slower aggregation and suppressed wormlike morphology observed in Figure 3.

### 2.5. Hydration of Polar Groups and the Shift in Packing Tendency

**Figure 5:**
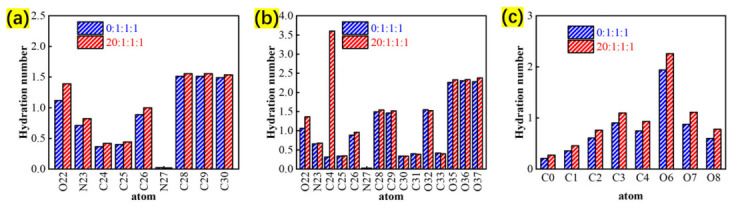
Hydration numbers of representative hydrophilic groups and Pts^−^ counterions in the calcium-free and Ca^2+^-containing systems. (**a**) Hydration numbers of representative polar atoms in EAHSB. (**b**) Hydration numbers of representative polar atoms in EHAC/EHA^+^. (**c**) Hydration numbers of representative atoms in Pts^−^. Blue and red bars represent the 0:1:1:1 and 20:1:1:1 systems, respectively.

The hydration analysis in Figure 5 provides a qualitative packing-parameter interpretation for why the Ca^2+^-containing systems favor smaller, higher-curvature aggregates. In the classical packing-parameter description, *p* = *v*/(*a*·*l*), where *a* is the effective hydrophilic headgroup area, *v* is the hydrophobic-tail volume, and *l* is the hydrophobic-tail length. The simulations show that Ca^2+^ increases the hydration numbers of the surfactant hydrophilic groups and the Pts^−^ counterion. A larger hydration shell increases the apparent hydrophilic area a and thereby reduces the apparent packing tendency toward low-curvature wormlike aggregates. This interpretation is also consistent with the broader role of hydration forces in colloidal stability and interfacial organization [10]. As shown in Figure 5a–c, the Ca^2+^-containing system generally exhibits higher hydration numbers around representative polar groups and Pts^−^ counterions. This indicates that more water molecules are retained near the micellar interfacial region after Ca^2+^ addition. Stronger hydration increases the apparent hydrophilic headgroup area. According to the packing-parameter model, an increase in the effective headgroup area decreases the packing parameter and favors higher interfacial curvature. As a result, low-curvature cylindrical or wormlike micelles become less favorable, whereas smaller high-curvature spherical aggregates become more stable. Thus, the hydration-number results directly connect the Ca^2+^-containing environment with the observed morphology shift.

### 2.6. Hydrophobic-Tail Exposure and Conformational Disorder

**Figure 6:**
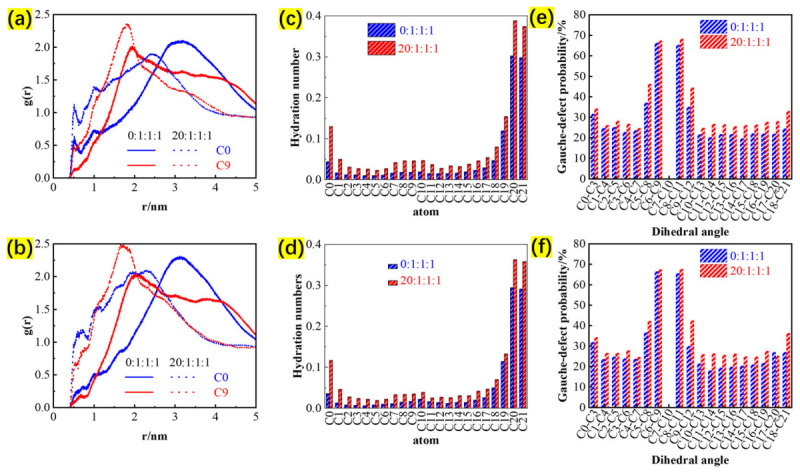
Hydrophobic-tail exposure and conformational disorder in the calcium-free and Ca^2+^-containing systems. (**a**,**b**) RDFs between surfactant quaternary ammonium nitrogen atoms and selected hydrophobic-tail carbon atoms for EHAC/EHA^+^ and EAHSB. (**c**,**d**) Hydration-number distributions along EHAC/EHA^+^ and EAHSB hydrophobic tails. (**e**,**f**) Gauche-defect probabilities along the hydrophobic tails of EHAC/EHA^+^ and EAHSB.

As shown in Figure 6a,b, the hydrophobic tails were not completely buried inside the micellar core. RDFs between the quaternary ammonium nitrogen and the terminal carbon C0 or double-bond carbon C8 showed that these tail carbons appeared near 0.4 nm from the headgroup. Thus, part of the tail population was located close to the micellar surface or oriented nearly parallel to it. In the Ca^2+^-containing system, the RDF peaks shifted to shorter distances, meaning that more tail segments approached the polar interface.

The hydration-number profiles of the tail carbons in Figure 6c,d support the same conclusion. Hydration was nonzero along the hydrophobic tails and followed a W-shaped profile: it decreased first, increased in the middle region, decreased again and reached a high value near the hydrophilic headgroup. C18-C21 showed stronger hydration because these atoms are closer to the polar headgroup region. The terminal carbon and the carbon atoms near the double bond were more hydrated than the middle carbons, indicating a greater tendency to approach the micellar surface. With Ca^2+^, the hydration numbers of the hydrophobic-tail carbons were higher than in the calcium-free system, which means that the hydrophobic core became less shielded from water.

The gauche-defect analysis in Figure 6e,f shows how this exposure is connected to chain packing. Low gauche probability corresponds to a more ordered all-trans-like chain, whereas a higher gauche probability indicates stronger bending and weaker packing. In both surfactants, the gauche probability decreased and then increased away from the double-bond region. The Ca^2+^-containing systems showed higher gauche-defect probabilities than the calcium-free system. These results indicate that Ca^2+^ is associated with increased tail conformational disorder, more frequent chain curling, and greater exposure of hydrophobic segments near the polar interface. The increased tail hydration and gauche-defect probability further explain the suppression of wormlike micelle growth. The formation of long wormlike micelles requires relatively compact and ordered hydrophobic-chain packing along the micellar axis. In the Ca^2+^-containing systems, higher tail hydration indicates greater exposure of hydrophobic segments to water, while higher gauche-defect probability reflects stronger chain bending and conformational disorder. These changes reduce the compactness and continuity of the hydrophobic core, making it more difficult for the aggregates to maintain elongated low-curvature wormlike morphology. Therefore, hydrophobic-tail disorder provides another structural reason for the shift toward smaller spherical aggregates.

### 2.7. Proposed Mechanism for Ca^2+^-Regulated Suppression of Wormlike Micelle Growth

Taken together, the trajectories point to a coupled mechanism rather than a single ion-screening event. In the calcium-free system, electrostatic association between EAHSB, EHAC and Pts^−^, together with hydrophobic-tail packing, drives fusion from small spherical micelles to elongated wormlike micelles. At sufficiently high local loading, these wormlike micelles extend through the periodic images and form a network-like structure. Such network-like aggregates are consistent with the microscopic aggregate features generally associated with viscoelastic surfactant fluids [1,2,5]. Compared with previously reported mixed ionic surfactant systems, the calcium-free EAHSB/EHAC/NaPts system follows a similar association-driven growth mechanism, in which charged-group association and counterion-mediated screening reduce the effective headgroup repulsion and promote low-curvature micellar growth. However, the Ca^2+^-containing systems show an ion-specific suppression behavior. Therefore, unlike the calcium-free EAHSB/EHAC/NaPts system, which follows association-driven wormlike micelle growth, the Ca^2+^-containing systems exhibit ion-specific suppression of one-dimensional aggregate growth.

In the Ca^2+^-containing systems, the suppression of wormlike micelle growth can be explained by a coupled mechanism supported by the above structural analyses. First, the RDF and coordination-number results show that several intermolecular charged-group correlations between EAHSB, EHAC, and Pts^−^ are weakened, indicating redistribution of the mixed interfacial charged layer. This weakens the continuous intermolecular association required for one-dimensional micellar growth. Second, the hydration-number results show that polar groups and Pts^−^ counterions become more strongly hydrated, which increases the apparent hydrophilic headgroup area and favors higher interfacial curvature. Third, the hydrophobic-tail hydration and gauche-defect analyses show that the hydrophobic core becomes less compact and more conformationally disordered. These coupled effects reduce the ability of aggregates to maintain elongated low-curvature morphology. Consequently, the Ca^2+^-containing systems show slower aggregate growth and a final morphology shifted from wormlike micelles to smaller spherical aggregates.

## 3. Materials and Methods

### 3.1. Force Field Parameterization

The zwitterionic surfactant EAHSB, cationic surfactant EHAC, and organic counterion NaPts were selected as the main components of the ionic viscoelastic surfactant system. Calcium ions were introduced to construct calcium-containing brine models and to examine how divalent ions regulate the self-assembly pathway and suppress wormlike micelle growth.

**Figure 7:**
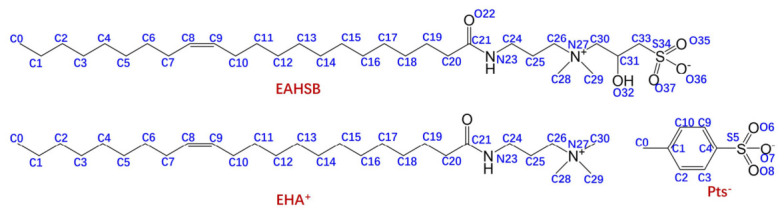
Molecular structures and atom labels of EAHSB, EHA^+^, and Pts^−^ used for force-field parameterization and molecular analysis.

The initial molecular structures of EAHSB, EHAC, and Pts^−^ were constructed according to their chemical structures, as shown in Figure 7. All-atom molecular dynamics simulations were performed within the CHARMM additive force-field framework. The bonded and non-bonded parameters of EAHSB, EHA^+^, and Pts^−^ were generated using the CHARMM General Force Field (CGenFF), which is designed for drug-like and organic molecules compatible with the CHARMM all-atom additive force fields [19,20]. Water molecules were described using the TIP3P water model [21]. The parameters for Na^+^, Ca^2+^, and Cl^−^ were taken from CHARMM-compatible ion parameters and used consistently with the selected water model [19]. Na^+^, Ca^2+^, and Cl^−^ ions were added according to the designed system compositions to maintain the required ionic environment and charge neutrality. For reproducibility, the atom types, partial charges, bonded parameters, and CGenFF penalty scores of EAHSB, EHA^+^, and Pts^−^ were checked before production simulations, and parameters with relatively high penalty scores were further inspected to ensure chemically reasonable bonded and non-bonded descriptions. The same CHARMM-compatible Na^+^, Ca^2+^, and Cl^−^ parameter set was used consistently with the TIP3P water model in all calcium-free and Ca^2+^-containing systems. The topology and parameter files generated for EAHSB, EHA^+^, and Pts^−^ are available from the corresponding author upon reasonable request.

### 3.2. Construction of Initial Simulation Systems

**Figure 8:**
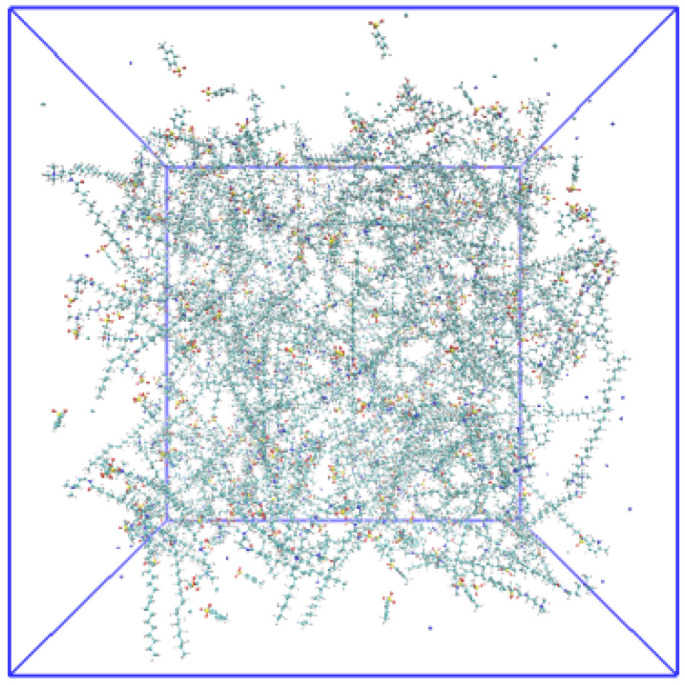
Initial random configuration of System 1 generated by Packmol before energy minimization. Water molecules, surfactants, and counterions were randomly distributed in the periodic simulation box.

The construction of the initial simulation boxes was performed with slight modifications according to the packing strategy reported by Martínez et al. [22]. As shown in Figure 8, Packmol (version 21.0.1) was used to randomly place surfactants, counterions, and water molecules into a periodic simulation box. For the self-assembly simulations, the molar ratio of EAHSB:EHAC:Pts^−^ was fixed at 1:1:1. To investigate the effect of local surfactant/counterion enrichment on micellar self-assembly, five systems with different total molecular loadings were constructed. Each system contained 30,000 water molecules, while the numbers of EAHSB, EHAC, and Pts^−^ molecules were set as 150, 165, 180, 225, and 275, respectively.

For the Ca^2+^-containing simulations, the numbers of EAHSB, EHA^+^, and Pts^−^ were fixed at 180 each, and Ca^2+^ was introduced at Ca^2+^:EAHSB:EHA^+^:Pts^−^ ratios of 0:1:1:1, 10:1:1:1, and 20:1:1:1. Na^+^ and Cl^−^ ions were added to maintain charge neutrality and the designed ionic composition. Because these finite simulation boxes represent local-enrichment states within micellar domains, the nominal concentrations should not be interpreted as direct macroscopic bulk concentrations. The purpose of these compositions was not to reproduce a specific bulk field formulation, but to construct controlled local-enrichment models for comparing how increasing Ca^2+^ abundance affects surfactant/counterion self-assembly. The 10:1:1:1 system was used to represent a Ca^2+^-rich local environment relevant to Ca^2+^-containing formation brines, whereas the 20:1:1:1 system was used to amplify the Ca^2+^ effect and identify the limiting suppression trend.

### 3.3. Molecular Dynamics Simulation Protocol

The molecular dynamics simulations were performed with slight modifications according to the standard GROMACS simulation workflow described by Abraham et al. [23]. All simulations were carried out using GROMACS (version 2025.3). Periodic boundary conditions were applied in all three spatial directions. Before the production simulations, each system was subjected to energy minimization using the steepest descent algorithm to remove unfavorable contacts and obtain a stable initial configuration.

During equilibration, the stability of the systems was monitored by checking thermodynamic properties, including potential energy, temperature, pressure, and density. Equilibration was considered complete when the potential energy and density exhibited relative fluctuations of less than 0.1% over a 10 ns window. These criteria were satisfied for all systems after the first 10 ns of NPT relaxation. After the initial 10 ns NPT relaxation, the potential energy and density of all systems fluctuated within 0.1% of their mean values over the subsequent 200 ns trajectory, indicating stable thermodynamic behavior before production-trajectory analysis. Production trajectories were analyzed after the systems showed stable thermodynamic behavior under the same simulation protocol. The production simulations were used to compare the relative self-assembly behavior and morphology evolution among different systems.

After energy minimization, the systems were first equilibrated under the NPT ensemble using the Berendsen weak-coupling method [24]. The same Berendsen method was then applied for temperature and pressure control during the production simulations. While the Berendsen thermostat does not rigorously reproduce the canonical ensemble, it was applied uniformly to all calcium-free and Ca^2+^-containing systems. Therefore, the observed relative trends in Ca^2+^-regulated morphology changes remain valid. All simulations were performed at 300 K and 1 atm. All bonds involving hydrogen atoms were constrained using the LINCS algorithm for non-water molecules and the SETTLE algorithm for water molecules. These constraints allow the use of a 2 fs integration time step without compromising numerical stability. In this study, to ensure conservative and robust sampling of the complex surfactant–counterion interactions, the integration time step was set to 0.001 ps (1 fs) throughout all production simulations. Long-range electrostatic interactions were calculated using the particle mesh Ewald method [25]. Lennard–Jones interactions were treated with a cutoff scheme. The production simulations were then performed to monitor concentration-dependent self-assembly and Ca^2+^-regulated morphological transitions. Molecular configurations and aggregate morphologies were visualized using VMD (version 1.9.3) [26].

### 3.4. Micellar Morphology Analysis

The micellar morphology analysis was performed with slight modifications according to previously reported molecular dynamics studies of wormlike micelles [15,16]. Simulation snapshots at different time points were extracted to monitor the self-assembly pathway of the EAHSB/EHAC/NaPts system. The morphological evolution from dispersed molecules to spherical aggregates, rod-like intermediates, and wormlike micelles was analyzed qualitatively from time-resolved snapshots. For the Ca^2+^-containing systems, time-resolved snapshots were used to evaluate the effect of Ca^2+^ on aggregation rate, final aggregate morphology, and suppression of wormlike micelle growth.

Periodic images were also visualized for high-loading systems to determine whether wormlike micelles formed extended or entangled network-like structures. In this study, micellar morphology was assigned mainly from time-resolved snapshots and supported by molecular-level structural analyses, including charged-group correlations, coordination numbers, hydration numbers, and hydrophobic-tail conformational descriptors. Quantitative shape descriptors such as radius of gyration, sphericity, shape factor, and principal moments of inertia were not used as primary criteria in the present work and should be included in future larger-scale morphology analyses.

### 3.5. Radial Distribution Function Analysis

The radial distribution function analysis was performed with slight modifications according to the method commonly used in surfactant and ion hydration simulations [11]. RDFs were calculated to characterize the local spatial arrangement of representative charged groups in the aggregates. The RDF between atom groups *i* and *j*, *g_ij_*(*r*), was calculated as:

(1) $$g_{ij}(r)=\frac{1}{4\pi r^{2}\rho_{j}}\frac{dn_{ij}(r)}{dr}$$

where *dn_ij_*(*r*) is the average number of atoms of type *j* located within a spherical shell between *r* and *r* + *dr* from atoms of type *i* and *ρ_j_* is the bulk number density of atom type *j*.

The analyzed charged groups included the sulfonate group of EAHSB, the quaternary ammonium group of EAHSB, the quaternary ammonium group of EHAC, and the sulfonate group of Pts^−^. RDFs between hydrophobic tail carbon atoms and quaternary ammonium groups were also calculated to evaluate whether hydrophobic chains were buried inside the micellar core or exposed near the polar interface.

### 3.6. Coordination Number Calculation

The coordination number analysis was performed with slight modifications according to RDF-based coordination calculations reported in previous molecular simulation studies [11]. The coordination number *N_ij_* between atom groups *i* and *j* was obtained by integrating the RDF curve up to the first minimum:

(2) $$N_{ij}=4\pi \rho_{j}\int_{0}^{r_{min}}g_{ij}(r)r^{2}dr$$

where *r_min_* is the position of the first minimum after the first RDF peak. The coordination numbers were used to quantify the average number of neighboring charged sites within the first coordination shell and to compare local association probabilities among different systems. Changes in coordination number in the Ca^2+^-containing systems were used to evaluate the redistribution and weakening of selected intermolecular charged-group correlations within the surfactant/counterion assemblies.

### 3.7. Hydration Number Analysis

The hydration number analysis was conducted with slight modifications according to previous simulations of ion-regulated surfactant hydration [11]. Water molecules located within the first hydration shell of the target group were counted according to the first minimum of the corresponding RDF between the target group and water oxygen atoms. Hydration numbers were calculated for the polar headgroups of EAHSB and EHAC, Pts^−^ counterions, and hydrophobic tail carbon atoms.

The hydration numbers of polar headgroups and counterions were used to evaluate changes in the effective headgroup area. The hydration distribution along hydrophobic tails was used to assess whether the tail segments were fully buried inside the micellar core or partially exposed to the aqueous phase. Increased hydration of hydrophobic tail atoms was interpreted as weakened hydrophobic packing and reduced micellar stability.

### 3.8. Gauche Defect Analysis of Hydrophobic Tails

The gauche defect analysis was performed with slight modifications according to conventional alkyl-chain conformational analysis in molecular dynamics simulations [27]. The dihedral angle distribution along surfactant hydrophobic tails was calculated to evaluate the conformational order of alkyl chains. A gauche defect was assigned when the dihedral angle deviated from the all-trans conformation by more than ±60°. The gauche defect probability was calculated along the hydrophobic tail carbon positions of EAHSB and EHAC.

A higher gauche defect probability indicated stronger chain bending, lower conformational order, and weaker hydrophobic packing. This analysis was used to clarify whether Ca^2+^-containing environments increased hydrophobic-tail disorder and thereby contributed to the suppression of low-curvature wormlike micelle growth.

### 3.9. Packing Parameter Interpretation

The interpretation of micellar morphology was performed with slight modifications according to the classical surfactant packing parameter theory proposed by Israelachvili et al. [9]. The packing parameter *p* was expressed as:

(3) $$p=\frac{v}{al}$$

where *v* is the hydrophobic tail volume, *a* is the effective headgroup area, and *l* is the hydrophobic tail length. According to classical surfactant packing theory, smaller packing parameters favor high-curvature spherical micelles, whereas intermediate packing parameters favor cylindrical or wormlike micelles [9]. In this study, changes in hydration number, charged-group association, and tail conformational disorder were used to explain how Ca^2+^ suppresses wormlike micelle growth and favors smaller spherical aggregates.

## 4. Conclusions

In this work, all-atom molecular dynamics simulations were used to investigate the calcium-regulated self-assembly of an EAHSB/EHAC/NaPts surfactant–counterion system. In the calcium-free systems, dispersed surfactant and counterion molecules first assembled into small spherical aggregates, which subsequently fused into larger micelles, rod-like intermediates, and finally wormlike micelles. Increasing the local surfactant/counterion loading further promoted one-dimensional micellar growth and network-like association. In contrast, the introduction of Ca^2+^ slowed the assembly process and shifted the final aggregate morphology from elongated wormlike micelles to smaller spherical aggregates. These results indicate that Ca^2+^ changes both the kinetic pathway and the morphological endpoint of surfactant/counterion self-assembly.

The suppression of wormlike micelle growth by Ca^2+^ arises from coupled molecular effects rather than simple electrostatic screening. RDF and coordination-number analyses show that Ca^2+^ redistributes local charged-group correlations between EAHSB, EHAC, and Pts^−^, weakening several intermolecular contacts that support mixed surfactant/counterion organization. Hydration analysis further indicates that Ca^2+^ enlarges the hydration shells of polar headgroups and counterions, increasing the effective hydrophilic area and shifting the packing tendency toward higher-curvature aggregates. Meanwhile, increased hydration and gauche-defect probabilities along the hydrophobic tails reveal looser chain packing and a less compact micellar core. Overall, the Ca^2+^-containing environment suppresses wormlike micelle growth through the coupling of charged-group redistribution, enhanced interfacial hydration, and hydrophobic-tail disorder, which weakens sustained one-dimensional micellar growth and favors smaller high-curvature spherical aggregates. These molecular insights provide guidance for designing salt-responsive or salt-tolerant viscoelastic surfactant fluids. Future work should combine longer-time and larger-scale simulations with robust quantitative morphology descriptors, such as radius of gyration, sphericity, shape anisotropy, shape factor, and principal moments of inertia, particularly for aggregates extending across periodic boundaries. Experimental validation using rheology, cryo-TEM, SANS/SAXS, or NMR measurements would further help connect molecular morphology with macroscopic viscoelastic behavior.

## Figures and Tables

**Table 1:** Molecular loadings used for the self-assembly simulations.

System	EAHSB	EHA^+^	Pts^−^	Cl^−^	Na^+^	H_2_O
1	150	150	150	150	150	30,000
2	165	165	165	165	165	30,000
3	180	180	180	180	180	30,000
4	225	225	225	225	225	30,000
5	275	275	275	275	275	30,000

**Table 2:** Composition and nominal Ca^2+^ molarity of Ca^2+^-containing simulation systems.

System	Ca^2+^:EAHSB:EHA^+^:Pts^−^	EAHSB	EHA^+^	Pts^−^	Ca^2+^	Na^+^	Cl^−^	H_2_O	Nominal Ca^2+^ Molarity/M
1	0:1:1:1	180	180	180	0	180	180	30,000	0
2	10:1:1:1	180	180	180	1800	180	3780	30,000	2.44
3	20:1:1:1	180	180	180	3600	180	7380	30,000	4.88

Note: The nominal Ca^2+^ molarity was estimated from the number of Ca^2+^ ions and the initial simulation-box volume of 10.7 × 10.7 × 10.7 nm^3^. These values describe finite-box local-enrichment states within micellar domains and should not be interpreted as direct macroscopic bulk formulation concentrations. The apparent concentrations in the simulation boxes are higher than typical bulk experimental concentrations because the finite systems are designed to represent locally enriched micellar domains and to distinguish Ca^2+^-regulated morphology trends within accessible simulation times.

**Table 3:** Coordination numbers between charged groups before and after Ca^2+^ addition.

Group Pair	0:1:1:1	20:1:1:1
EAHSB_S-EAHSB_S	0.97 ± 0.03	0.72 ± 0.07
EAHSB_S-EAHSB_N	2.95 ± 0.07	3.62 ± 0.09
EAHSB_S-EHAC_N	1.69 ± 0.05	1.40 ± 0.01
EAHSB_S-Pts_S	1.08 ± 0.02	0.92 ± 0.03
EAHSB_N-EAHSB_N	2.20 ± 0.10	1.76 ± 0.07
EAHSB_N-EHAC_N	2.20 ± 0.09	1.81 ± 0.08
EAHSB_N-Pts_S	1.81 ± 0.06	1.84 ± 0.08
EHAC_N-EHAC_N	1.90 ± 0.02	1.55 ± 0.04
EHAC_N-Pts_S	2.46 ± 0.11	2.16 ± 0.06
Pts_S-Pts_S	0.41 ± 0.04	0.05 ± 0.00

## Data Availability

The data presented in this study are available from the corresponding author upon reasonable request.
